# Outpatient vs. inpatient hip arthroplasty: a matched case-control study on a 90-day complication rate and 2-year patient-reported outcomes

**DOI:** 10.1186/s13018-020-01871-8

**Published:** 2020-08-31

**Authors:** Philip J. Rosinsky, Sarah L. Chen, Mitchell J. Yelton, Ajay C. Lall, David R. Maldonado, Jacob Shapira, Mitchell B. Meghpara, Benjamin G. Domb

**Affiliations:** 1 American Hip Institute Research Foundation, Des Plaines, IL 60018 USA; 2 Sidney Kimmel Medical College, Philadelphia, PA 19107 USA; 3 American Hip Institute, Des Plaines, IL 60018 USA; 4 AMITA Health St. Alexius Medical Center, Hoffman Estates, IL 60169 USA

**Keywords:** Total hip arthroplasty, Outpatient arthroplasty, Patient-reported outcomes

## Abstract

**Background:**

The transition to outpatient-based surgery is a major development occurring in recent years in the field of total hip arthroplasty (THA). The effect of this transition on patient-reported outcomes (PROs) is still not well established. The purpose of the current study was to compare patients undergoing inpatient THA (iTHA) to patients undergoing outpatient THA (oTHA) regarding (1) perioperative variables including surgical time, blood loss, and length of stay (2) 90-day complication rates and unplanned emergency room or office visits (3) 2-year PROs including modified Harris hip score (mHHS), Harris hip score (HHS), forgotten joint score (FJS), pain, and satisfaction, as well as the quality of live measures.

**Methods:**

The American Hip Institute registry was analyzed for patients undergoing THA between July 2014 and April 2016. The first 100 patients undergoing oTHA were selected and matched to 100 patients undergoing iTHA via propensity matching based on the following variables: age, sex, body mass index (BMI), Charlson comorbidity index (CCI), and smoking status. The primary outcomes were PROs at 2 years post-operatively. The secondary outcomes were perioperative surgical variables, 90-day complication rates, and unplanned emergency and clinic visits.

**Results:**

After exclusions, 91 patients remained in each group and were compared. The oTHA group showed improved 2-year PROs with regard to mHHS (91.5 vs. 86.2; *P* = 0.02), HHS (92.3 vs. 87.4; *P* = 0.02), and pain (1.0 vs. 1.5; *P* = 0.04). The oTHA group had an average length of stay of 6.8 h compared to 43.2 h for the iTHA group (*P* < 0.001). There were no significant differences between the groups regarding readmissions, emergency room visits, and unplanned clinic visits. Complications and revision rates were similar in both groups.

**Conclusion:**

In appropriately selected, younger patients, oTHA can achieve improved postoperative 2-year PROs compared to iTHA. We found no differences regarding postoperative short-term complications or 2-year revision rates, and no differences in unplanned office visits or readmissions.

**Level of evidence:**

Prognostic level 3.

## Introduction

Total hip arthroplasty (THA) is one of the most common and successful surgeries in orthopedics, with a 2007 Lancet article dubbing it “the operation of the century.” [1] Further, the demand for THA is growing, given the world’s aging population and consequential rise in osteoarthritis [2]. Over the last decade, major developments have been occurring throughout the orthopedic field, and specifically in the realm of arthroplasty, including the transition to fast-track and outpatient-based surgery, as well as the introduction of navigational and robotic-assisted surgeries.

Studies show that THA is shifting to the outpatient setting worldwide, with estimates showing that by 2026, more than half of THAs in the USA will be performed as an outpatient procedure [3, 4]. This transition has been enabled by various factors including the rise in multidisciplinary care coordination, standardization of perioperative protocols, and development of rapid rehabilitation protocols [5–7]. As the main concern of outpatient THA is patient safety, several studies have investigated immediate and delayed postoperative complications, with a systematic review finding equivalent complications between outpatient and inpatient arthroplasty [8].

There is limited literature on PROs comparing outpatient THA (oTHA) and inpatient THA (iTHA). The purpose of this study was to compare prospectively collected 2-year outcomes and 90-day complications in 100 consecutive patients undergoing oTHA with a matched cohort of 100 patients undergoing iTHA. Our hypothesis was that patients undergoing outpatient surgery would demonstrate higher postoperative PROs, while not incurring higher complication or revision rates.

## Methods

The first 100 consecutive patients who underwent oTHA at our institution were group matched to 100 patients who underwent iTHA during the same time period: July 2014 to April 2016. Outpatient cases were matched to inpatient cases with similar age, sex, BMI, laterality, approach, Charlson Comorbidity Index (CCI), and smoking status via propensity score matching.

The CCI was used to assess comorbid diseases in the outpatient and inpatient groups. This index generates a numerical score incorporating the following variables: age, myocardial infarction, chronic heart failure (CHF), peripheral vascular disease, cerebrovascular accident (CVA), dementia, chronic obstructive pulmonary disease (COPD), connective tissue disease, peptic ulcer disease, liver disease, diabetes mellitus, hemiplegia, chronic kidney disease (CKD), cancer, and AIDS [9]. In addition, the American Society of Anesthesiologists (ASA) Score was used to assess the physical status of patients before surgery. ASA scores were defined as follows: 1: normal healthy patient, 2: patient with mild systemic disease, 3: patient with severe systemic disease, and 4: patient with severe systemic disease that is a constant threat to life [10]. In addition, smoking status was recorded as it has been shown to increase the risk of inpatient complications, costs, and length of stay following THA [11–13].

A total of 8 patient-reported outcomes (PROs) were provided for both groups: Harris Hip Score (HHS), modified Harris Hip Score (mHHS), Forgotten Joint Score (FJS), the physical and mental components of the Veterans Rand-12 Item Health Survey (VR-12P and VR-12M, respectively), the physical and mental components of the Short Form (SF-12P and SF-12M, respectively), and a visual analog pain scale (VAS) for pain. In addition, satisfaction with surgery was reported, with 0 being the least satisfied and 10 being the most satisfied. For FJS, the proportion of patients attaining the previously published “successful” threshold was determined for each group [14]. Perioperative outcomes were collected including surgical time and blood loss, length of hospitalization, and radiographic placement of components. In addition, unplanned office visits in the first 90 days following surgery, revision surgeries, and complications were noted for both groups. Complications were classified according to the Clavien-Dindo classification system [15, 16].

In order to detect an 8-point difference in HHS, a priori power analysis indicated a sample size of 82 in each group was necessary to detect statistical significance between groups with at least 80% power [17]. In order to allow for exclusions and loss to follow-up, an initial group size of 100 patients was chosen.

All statistical analysis was performed using Microsoft Excel (Microsoft Corporation; Redmond, WA) and the Real Statistics Add-In. Data was assessed for normality using the Shapiro-Wilk Test and was assessed for equal variance using the *F* test. Normally distributed data sets were compared using the Student’s *T* test. The Mann-Whitney and Welch test were used to compare non-normally distributed data with equal and unequal variances, respectively. Categorial variables were assessed with the chi-square test or Fisher’s exact test. The threshold for statistical significance was set at *P* < 0.05.

While the present study represents a unique analysis, data on some patients in this study has been reported in other studies. All data collection received Institutional Review Board approval.

### Indications for outpatient THA

All patients underwent a comprehensive physical examination and radiographic evaluation by the senior surgeon (BGD). At our institution, beginning in 2014, all patients without significant comorbidities were offered the option of outpatient THA, and the decision whether to proceed with an outpatient procedure was based on patient preference. All patients had hip osteoarthritis which impaired their activities of daily living and were refractory to a minimum of 3 months of conservative treatment (rest, activity modification, physical therapy, and non-steroidal anti-inflammatory drugs).

### Surgical technique

Preoperative planning based on anteriorposterior (AP) plain radiography using the TraumaCad software (Brainlab, Munich, Germany) was performed. Using the MAKO^TM^ robotic-arm-assisted total hip system (Stryker Orthopaedics, Mahwah, NJ), an additional 3-dimensional model was built during preoperative planning based on computed tomography (CT) scans. Prior to surgery, the senior surgeon (BGD) used this model to facilitate surgical planning and to achieve restoration of patient-specific leg length and global offset.

The preferred surgical approach was the direct anterior approach; although, patients with an associated gluteus medius tear or in the need of hardware removal underwent a THA with the posterior approach. On the day of surgery, general anesthesia and intravenous tranexamic acid (10 mg/kg) were administered prior to incision. The operative hip was prepared and draped in a sterile fashion. After performing the capsulotomy, femoral registration was performed followed by a robotic-assisted femoral neck cut. Following removal of the femoral head, acetabular exposure was completed, registration of the acetabulum was performed, and then the acetabulum was reamed using robotic guidance. Using the robotic arm, the appropriate acetabular cup implant and liner were impacted into place. The femur was then broached, and with femoral trials in place, a clinical evaluation of stability and navigation confirmation of stem version, leg length discrepancy (LLD), and offset, was performed. The femoral stem and head were implanted and a final confirmation of LLD, offset, and stability was recorded. The wound was closed by layers and a drain was placed. All THAs were performed using a cementless cup with polyethylene liner (Trinity; Corin, UK), uncemented stem (Metafix; Corin, UK), and a ceramic head (Biolox Delta; CeramTec, Plochingen, Germany).

### Postoperative rehabilitation

Patients in both cohorts were transferred to recovery suites. Patients in the outpatient cohort were discharged after medical stabilization, pain management, and initiation of ambulation as soon as the patient was alert and showed adequate stability and strength. Patients in the inpatient cohort were admitted at least for one night, after which they were similarly discharged, if medically stable.

The majority of patients were discharged home (> 90%) with a minority discharged to skilled nursing facilities (< 10%). Patients continued physical therapy for six to eight additional weeks and had follow-up appointments at 2 weeks, 1 month, and annually thereafter. PROs and complications were collected at the preoperative visit and each subsequent postoperative follow-up visit.

## Results

### Demographics

**Fig. 1:**
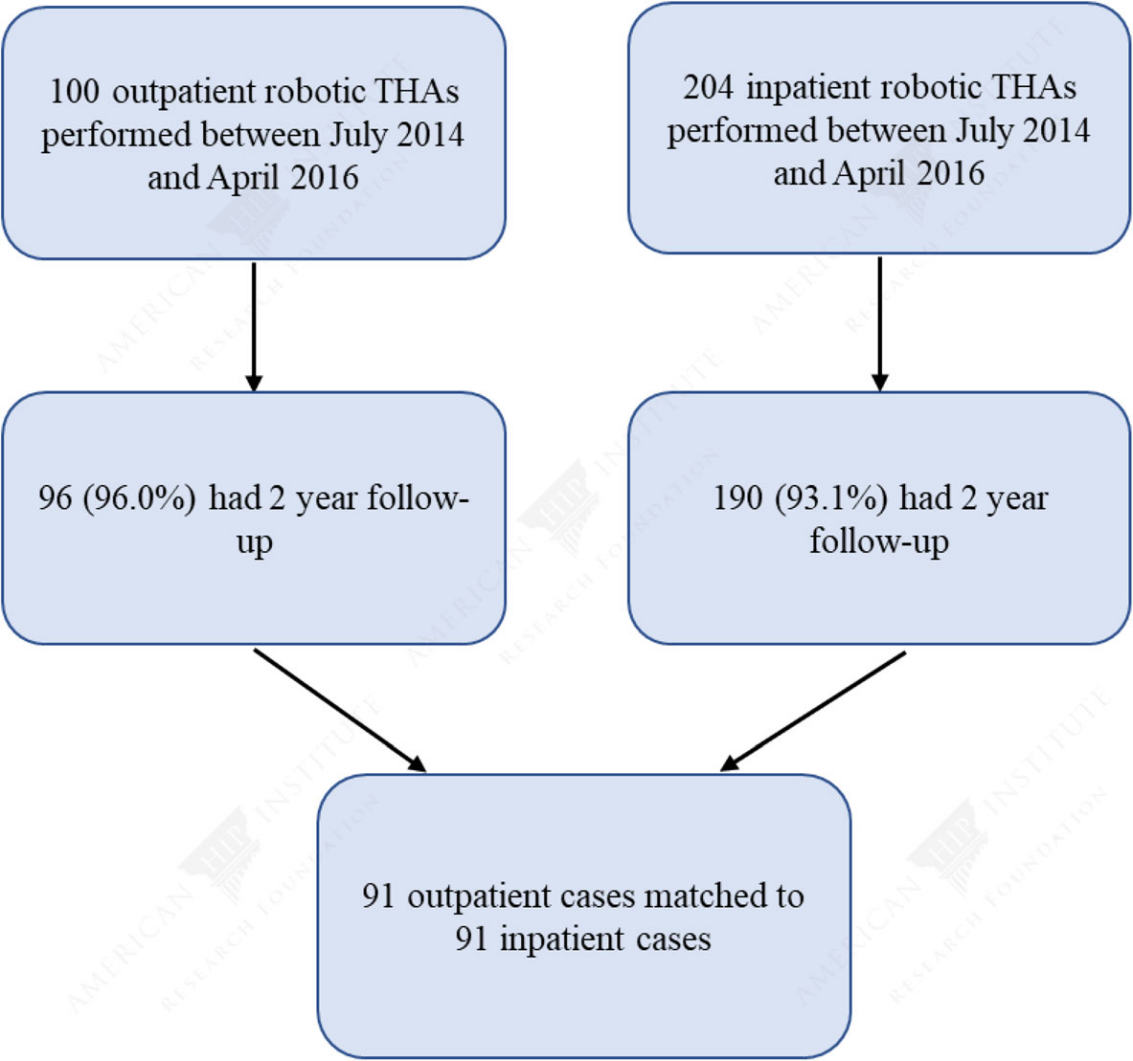
Patient selection flow diagram. THA: total hip arthroplasty

Our patient selection process is illustrated in Fig. 1. Following the matching process, 91 outpatient cases were matched to 91 inpatient cases. In the outpatient group, there were 38 (41.8%) females and 53 (58.2%) males, and in the inpatient group, there were 42 (46.2%) females and 49 (53.8%) males (*P* = 0.654). In addition, there were no significant differences in age or BMI between the two groups (Tables 1 and 2). In the outpatient group, there were 7 current smokers, and in the inpatient group, there were 8 current smokers (*P* = 0.788). With regard to Charlson Comorbidity Index, there was a similar distribution between the outpatient and inpatient groups (*P* = 0.984). In both groups, there were 9 patients with a CCI ≥ 3 and the majority of patients had an ASA score of 2. There was no significant difference in surgical time or blood loss between the two groups (*P* > 0.05).

**Table 1:** Summary demographics for both groups

	Outpatient	Inpatient	*P* value
Hips included in study	91	91	
Left	44 (48.4%)	43 (47.3%)	0.882
Right	47 (51.6%)	48 (52.7%)	
Gender			
Female	38 (41.8%)	42 (46.2%)	0.654
Male	53 (58.2%)	49 (53.8%)	
Approach			
Anterior	87 (95.6%)	86 (94.5%)	0.732
Posterior	4 (4.4%)	5 (5.5%)	
Age at surgery (years, mean, SD, range)	53.3 ± 7.2 (29.6- 65.8)	55.3 ± 6.9 (36.9 - 75.8)	0.12
BMI (kg/m^2^, mean, SD, range)	29.2 ± 5.2 (15.4- 44.3)	29.4 ± 5.8 (20.6- 47.8)	0.568
Charlson Comorbidity Index, *n*			
0	21 (23.1%)	20 (22.0%)	0.984
1–2	61 (67.0%)	62 (68.1%)	
≥ 3	9 (9.9%)	9 (9.9%)	

*SD* standard deviation, *BMI* body mass index, *n* sample size, *mo* months

**Table 2:** Intraoperative data for both groups

	Outpatient	Inpatient	*P* value
Follow-up (mo, mean, SD)	34.9 ± 7.2 (24.0–55.6)	37.8 ± 8.5 (24.0–57.6)	0.019
ASA Score^1^			0.076
1	14 (16.5%)	18 (20.0%)	
2	61 (71.8%)	51 (56.7%)	
3	10 (11.8%)	21 (23.3%)	
Surgical time (min, mean, SD)	113.6 ± 30.1	116.1 ± 27.4	0.794
Blood loss (mL, mean, SD)	338.7 ± 187.6	396.1 ± 242.1	0.083
Hours at surgery center/hospital (hours, mean, SD)	6.8 ± 1.4	43.2 ± 20.3	< 0.001
Cup inclination (mean, SD)	41.0 ± 3.4	40.7 ± 3.3	0.543
Cup version (mean, SD)	19.5 ± 4.3	19.5 ± 3.8	0.971
Leg length discrepancy (mm, mean, SD)	0.13 ± 3.0	0.96 ± 3.3	0.162
Global offset (mm, mean, SD)	72.5 ± 7.6	73.6 ± 6.0	0.288

^1^ASA score only available for 85 outpatient cases and 90 inpatient cases

*SD* standard deviation

### Patient-reported outcomes

**Fig. 2:**
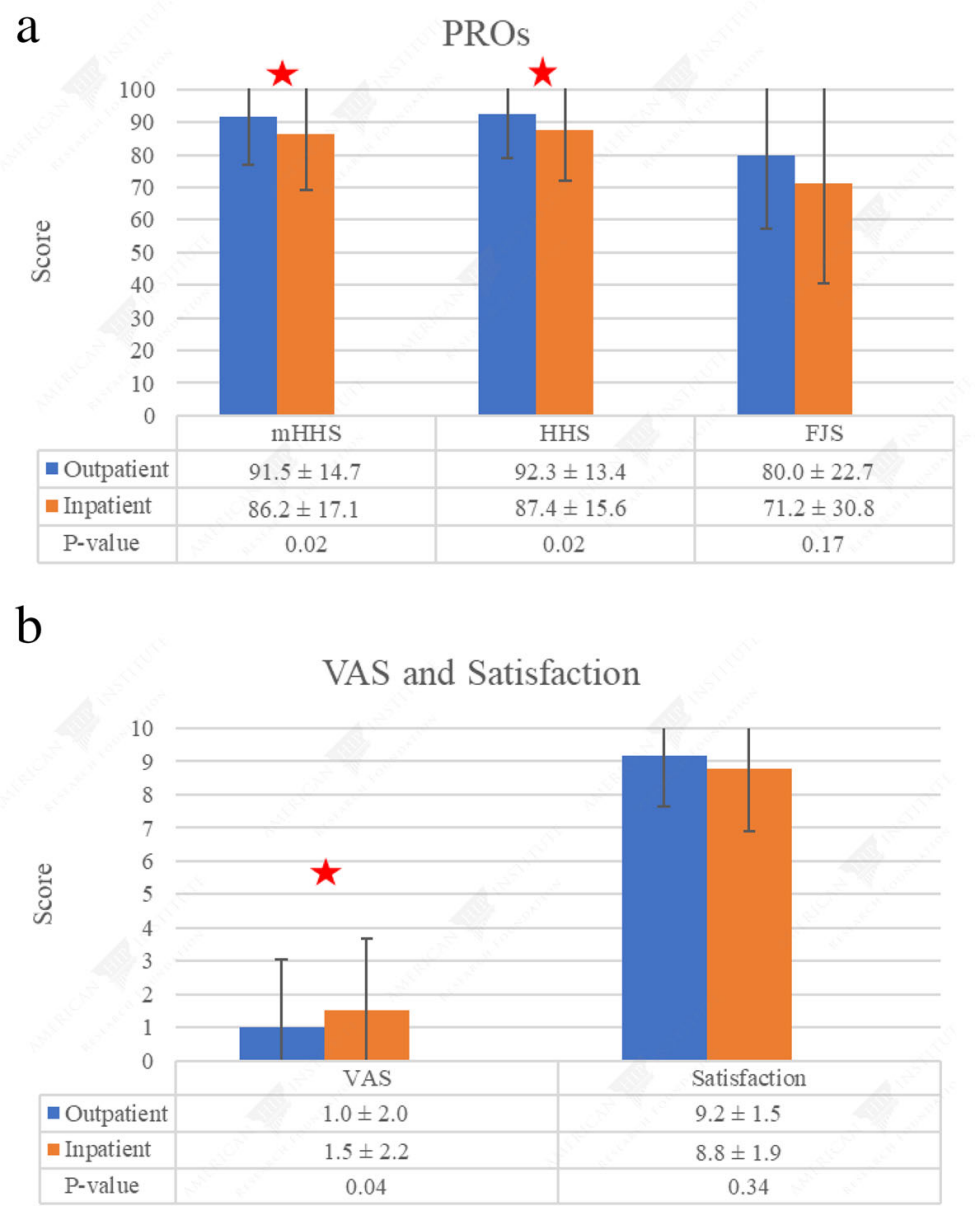
**a** Mean ± SD, mHHS: modified Harris Hip Score, HHS: Harris Hip Score, FJS: Forgotten Joint Score. **b** Mean ± SD, VAS: visual analog scale

The mean preoperative mHHS scores for the outpatient and inpatient groups were 15.3 ± 5.7 and 16.9 ± 9.1, respectively (*P* = 0.591). There was no significant difference in preoperative PROs or preoperative pain (VAS) between the two groups (Table 3). Two-year postoperative PROs for the outpatient and inpatient groups are summarized in Table 4. Outpatient cases demonstrated significantly higher mHHS and HHS scores at latest follow-up (91.5 ± 14.7 vs. 86.2 ± 17.1, *P* = 0.023 and 92.3 ± 13.4 vs. 87.4 ± 15.6, *P* = 0.023, respectively) (Fig. 2a). With regard to FJS, VR-12M, VR-12P, SF-12M, and SF-12P scores, there was no significant difference between the two groups (*P* > 0.05). Outpatient cases experienced significantly less pain than inpatient cases (1.0 ± 2.0 vs 1.5 ± 2.2, *P* = 0.0434) at 2 years postoperatively (Fig. 2b). The mean satisfaction with surgery was 9.2 out of 10 in the outpatient group and 8.8 out of 10 in the inpatient group (*P* = 0.336).

**Table 3:** Preoperative patient-reported outcomes for both groups

Patient-reported outcome	Outpatient	Inpatient	*P* value
mHHS (mean, SD, range)	15.3 ± 5.7 (10–30)	16.9 ± 9.1 (0–44)	0.591
HHS (mean, SD, range)	14.8 ± 2.1 (9–18.1)	13.7 ± 2.7 (9–18.1)	0.057
VR-12 M (mean, SD, range)	32.3 ± 7.5 (20.6–53.4)	30.0 ± 7.8 (14.9–52.8)	0.168
VR-12P (mean, SD, range)	53.7 ± 10.9 (30.7–65.9)	53.2 ± 10.6 (25.4–67.6)	0.756
SF-12 M (mean, SD, range)	48.4 ± 13.6 (20–78)	48.2 ± 18.7 (0–83)	0.837
SF-12P (mean, SD, range)	52.6 ± 11.1 (27.7–66.2)	51.1 ± 11.4 (26.3–66.2)	0.522
VAS (mean, SD, range)	6.4 ± 2.3 (0–10)	5.2 ± 3.0 (0–10)	0.057

*SD* standard deviation, *mHHS* modified Harris Hip Score, *HHS* Harris Hip Score, *VR-12M* Veterans RAND 12-Item Health Survey Mental, *VR-12P* Veterans RAND 12-Item Health Survey Physical, *SF-12M* Short Form 12 Mental, *SF-12P* Short Form 12-Physical

**Table 4:** Two-year patient-reported outcomes for both groups

Patient-reported outcome	Outpatient	Inpatient	*P* value
mHHS (mean, SD, range)	91.5 ± 14.7 (35.0–100)	86.2 ± 17.1 (30.0–100)	0.023
HHS (mean, SD, range)	92.3 ± 13.4 (40.9–100)	87.4 ± 15.6 (36.3–100)	0.023
FJS (mean, SD, range)	80.0 ± 22.7 (18.8–100)	71.2 ± 30.8 (0–100)	0.169
VR-12 M (mean, SD, range)	62.1 ± 5.5 (43.6–69.7)	60.4 ± 8.0 (23.0–69.7)	0.156
VR-12P (mean, SD, range)	51.4 ± 8.9 (13.9–59.5)	48.9 ± 10.6 (14.6–60.3)	0.121
SF-12 M (mean, SD, range)	57.7 ± 5.4 (34.6–69.4)	56.3 ± 7.9 (22.9–70.5)	0.375
SF-12P (mean, SD, range)	49.8 ± 9.5 (16.1–58.6)	47.4 ± 11.0 (17.0–58.8)	0.132
VAS (mean, SD, range)	1.0 ± 2.0 (0–8.44)	1.5 ± 2.2 (0–10)	0.043
Satisfaction (mean, SD, range)	9.2 ± 1.5 (2–10)	8.8 ± 1.9 (0–10)	0.336

*SD* standard deviation, *mHHS* modified Harris Hip Score, *HHS* Harris Hip Score, *FJS* Forgotten Joint Score, *VR-12M* Veterans RAND 12-Item Health Survey Mental, *VR-12P* Veterans RAND 12-Item Health Survey Physical, *SF-12M* Short Form 12 Mental, *SF-12P* Short Form 12-Physical

### Revisions, complications, and unplanned clinic visits

The number of complications as classified according to the Clavien-Dindo system is provided in Table 5. There was no significant difference in the type of complications experienced in both groups (*P* = 0.211). Two patients (2.2%) in the inpatient group underwent a revision THA for acetabular cup loosening at 1.5 and 2 years after the index surgery. There were no patients in the outpatient group that required a revision THA. All outpatient cases were discharged home the day of surgery. For the inpatient group, 84 (92.3%) were discharged home and 7 (7.7%) were discharged to an extended care facility or rehabilitation center. The total number of unplanned office visits and ER visits is detailed in Table 6.

**Table 5:** Complications for both groups

Complications	Outpatient	Inpatient	*P* value
Clavien-Dindo Classification			0.211
I	**10 (11.0%)**	**10 (11.0%)**	
Wound dehiscence	6 (6.6%)	1 (1.1%)	
Periprosthetic/calcar fracture	2 (2.2%)	–	
Greater trochanteric fracture	–	1 (1.1%)	
Iliopsoas tendinitis	–	1 (1.1%)	
Urinary retention/constipation	–	3 (3.3%)	
Rash	2 (2.2%)	1 (1.1%)	
Allergic reaction to narcotics	–	1 (1.1%)	
Sciatica	–	1 (1.1%)	
Psychiatric issues	–	1 (1.1%)	
II	**1 (1.1%)**	**4 (4.4%)**	
Urinary tract infection	1 (1.1%)	1 (1.1%)	
Anemia requiring transfusion	–	2 (2.2%)	
ER visit for pain management	–	1 (1.1%)	
IIIa	**0**	**3 (3.3%)**	
Incision revision	–	3 (3.3%)

**Table 6:** Unplanned Office/ER Visits

Unplanned office/ER visits	Outpatient	Inpatient	*P* value
Unplanned office visits	9 (9.9%)	3 (3.3%)	0.135
ER visits	2 (2.2%)	2 (2.2%)	> 0.999

*ER* emergency room

## Discussion

Compared to an inpatient group with similar age, sex, BMI, laterality, approach, Charlson Comorbidity Index (CCI), and smoking status, the outpatient group demonstrated superior outcomes at minimum 2 years postoperatively. There were significant differences in mHHS (91.5 vs. 86.2), HHS (92.3 vs. 87.4), and pain (1.0 vs. 1.5) at the latest follow-up. In addition, although not statistically significant, the outpatient group exhibited higher FJS at the latest follow-up (80.0 vs. 71.2, *P* = 0.17). Existing literature on outpatient THA focuses on perioperative patient safety as manifested in 90-day complication rates and readmissions. To date, there is only one study that directly compares inpatient and outpatient THA outcomes, and PROs were analyzed at 4-weeks postoperatively [18]. To our knowledge, this is the first study to compare 2-year PROs between an outpatient and inpatient THA group.

As the patient populations were matched for multiple variables, the reason for the improved outcomes in the outpatient population warrants discussion. One possibility that needs to be considered is the socioeconomic background of the patients. It is possible that despite the matching process, the inpatient population may have had a less robust home-based support system or been of lower socioeconomic status. Social support, including having someone to discuss concerns, reinforce goals, and provide daily care, can increase a patient’s self-efficacy during the sensitive postoperative period [19, 20]. In addition, socioeconomic status has been shown to influence both immediate and long-term outcomes following THA [21–23]. Another possibility may be the satisfaction with the surgical experience as this has been shown to influence post-surgical PROs [24]. Lastly, the length of stay was significantly longer in the inpatient group, at nearly 2 days. This may influence the immediate postoperative course, including sleep quality, nutrition, and comfort levels, which may in turn impact long-term outcomes.

There were no significant differences in ASA scores, length of surgery, and blood loss between the two groups. The reduction in length of hospital stay in the outpatient group was very significant (6.8 h vs. 43.2 h, *P* < 0.001). Based on the 2017 American Hospital Association (AHA) Annual Survey, the average expense per inpatient day in the USA is $2,424 [25]. Applying this data to our study, performing THA on an outpatient basis represents a savings of $3,676 per patient.

Several studies have reported a > 90% same-day discharge rate in patients undergoing outpatient THA [5, 26, 27]. A systematic review on 955 patients undergoing outpatient total joint arthroplasty (TJA) found that 94.7% of patients were discharged the same day [28]. In this study, all patients in the outpatient group met the discharge criteria and were discharged home the day of surgery, as planned. Same-day discharge has been facilitated by a number of factors, including proper patient selection for outpatient surgery, comprehensive preoperative patient education, and an optimized perioperative rapid recovery protocol.

In addition, we evaluated readmissions, visits to the emergency room, and unplanned clinic visits. We did not detect any statistically significant differences in these measures, although this study may have been underpowered in this respect. There were no 90-day readmissions in the outpatient group, and there was one readmission for wound revision in the inpatient group. In a study on 250,000 THAs using the Nationwide Readmissions Database, the rates of 30- and 90-day readmissions after THA were 4% and 8%, respectively. The authors found that a patient’s length of stay had the greatest influence on the cost of 90-day readmissions [29]. In studies on outpatient TJA, rates of 90-day readmission are very low, at approximately 0.5–1% [28, 30].

There are mixed findings on whether performing TJA on an outpatient basis increases the burden on the surgeon. Shah et al. found that outpatient TJA shifts the burden of care from the hospital to the surgeon, as outpatient surgery requires increased preoperative patient education and results in more patient phone calls the week following surgery [31]. Conversely, in a multi-center, prospective, randomized study, Goyal et al. found no significant difference in the outpatient and inpatient THA group with regard to calls between the office staff and patients [18].

The primary concern in performing outpatient THA has been patient safety and complications. Based on the findings in this study, there were no significant differences between the two groups in complication rates. In several studies, in appropriately selected patients, complications have been shown to be equal or lower in patients undergoing outpatient THA. Courtney et al. analyzed the American College of Surgeons-National Surgical Quality Improvement Program (ACS-NSQIP) database and found an 8% complication rate in the outpatient setting compared to a 16% complication rate in the inpatient setting [30]. Arshi et al. performed a registry-based study on 2184 patients undergoing outpatient THA and found comparable rates of surgical and medical complications [32].

It is noteworthy that seven patients in both groups combined experienced wound healing complications, although without further deep infections. All patients improved with local wound care and oral or topical antibiotics. The relatively higher rate of wound complications in this series can be explained by the predominant utilization of the direct anterior approach in patients included in this study. Multiple studies have demonstrated increased rates of wound complication when comparing the direct anterior approach to other surgical approaches [33–36].

There are multiple notable strengths in the present study. First, outpatient cases were matched to a contemporary group of inpatient cases with similar age, sex, BMI, laterality, approach, Charlson Comorbidity Index (CCI), and smoking status. Second, the patients included in the outpatient cohort were a prospectively selected, consecutive series of patients which enabled consistent data acquisition, and reduced selection bias. Third, this study analyzed multiple outcomes, including several PROs, readmissions, 90-day complications, unplanned office visits, and a 2-year revision rate. Fourth, by analyzing a single surgeon’s patient population, performance bias may be minimized enabling direct comparison between the inpatient and outpatient setting. This consistency is evident in the almost identical radiographic outcomes following surgery.

This study is not without limitations. Our study population included a younger than average cohort, with the average age being 53 and 55 for the outpatient and inpatient groups, respectively. Studies on outpatient THA have tended to consist of patients that are younger than the general population undergoing THA [28]. In a multi-national study on nearly 500,000 THA procedures, more than 60% of patients were aged 60–79, with only 13–20% belonging to the 50–59 age group [37]. This discrepancy is multifactorial, stemming from the senior author’s practice, which includes a younger, more active patient population, and the inherently younger cohort of inpatient THA cases needed to match the age of the outpatient THA group. Therefore, conclusions from this study may not be applicable to an older, more morbid patient population.

Second, the choice to undergo an inpatient or an outpatient procedure is determined by multiple factors, several of which depend on patient comorbidities. For the purpose of this study, we utilized a propensity-score matching process that consisted of several variables including medical comorbidities, smoking status, age, and BMI. Despite this matching process, the inpatient group had more patient graded ASA 3, although this was not statistically significant.

Additionally, this study consists of a mixed group of anterior and posterior THAs. However, in both groups, the patients undergoing posterior THA represented a small minority (4.4% and 5.5% for inpatient and outpatient cohorts, respectively).

Fourth, although we addressed postoperative follow-up in readmissions, emergency room visits, and unplanned office visits, we did not account for additional phone calls between the office staff and patients. This was inconsistently recorded and therefore was omitted from our analysis.

Lastly, this study was based on a single surgeon’s practice, which may limit the generalizability of our results. This practice is a high-volume practice, with extensive experience operating in the outpatient setting, including arthroscopic and arthroplasty procedures. The workflow developed by the surgeon and the office staff to optimize outpatient management may be difficult to apply to smaller practices.

## Conclusion

In conclusion, in appropriately selected, younger patients, outpatient THA can achieve improved postoperative patient-reported outcomes compared to inpatient THA. At 90-days postoperatively, we found similar readmission rates, unplanned office and emergency room visits, and complication rates between the two groups. At 2 years postoperatively, no significant differences were found in revision rates.

## Data Availability

The datasets analyzed during the current study come from the American Hip Institute Hip Preservation Registry and are available from the corresponding author on reasonable request.

## Abbreviations

THA: Total hip arthroplasty
PRO: Patient-reported outcome
iTHA: Inpatient total hip arthroplasty
oTHA: Outpatient total hip arthroplasty
mHHS: Modified Harris Hip Score
HHS: Harris Hip Score
FJS: Forgotten Joint Score
BMI: Body mass index
CCI: Charlson comorbidity index
CHF: Chronic heart failure
CVA: Cerebrovascular accident
COPD: Chronic obstructive pulmonary disease
CKD: Chronic kidney disease
ASA: American Society of Anesthesiologists
VR-12: Veterans Rand-12 item Health Survey
SF-12: Short Form-12
VAS: Visual analog scale
AP: Anterioposterior
CT: Computed tomography
LLD: Leg length discrepancy
TJA: Total joint arthroplasty

## References

[1] Learmonth ID, Young C, Rorabeck C (2007). The operation of the century: total hip replacement. Lancet Lond Engl.

[2] Sloan M, Premkumar A, Sheth NP (2018). Projected volume of primary total joint arthroplasty in the U.S., 2014 to 2030. J Bone Joint Surg Am.

[3] DeCook CA. Outpatient joint arthroplasty: transitioning to the ambulatory surgery center. J Arthroplast. 2019;S0883540319300336. 10.1016/j.arth.2019.01.006 30773355

[4] Argenson J-NA, Husted H, Lombardi A, Booth RE, Thienpont E (2016). Global forum: an international perspective on outpatient surgical procedures for adult hip and knee reconstruction. J Bone Jt Surg.

[5] Berger RA, Jacobs JJ, Meneghini RM, Della Valle C, Paprosky W, Rosenberg AG (2004). Rapid rehabilitation and recovery with minimally invasive total hip arthroplasty. Clin Orthop.

[6] Stambough JB, Nunley RM, Curry MC, Steger-May K, Clohisy JC (2015). Rapid recovery protocols for primary total hip arthroplasty can safely reduce length of stay without increasing readmissions. J Arthroplast.

[7] Yanik JM, Bedard NA, Hanley JM, Otero JE, Callaghan JJ, Marsh JL (2018). Rapid recovery total joint arthroplasty is safe, efficient, and cost-effective in the veterans administration setting. J Arthroplast.

[8] Pollock M, Somerville L, Firth A, Lanting B. Outpatient total hip arthroplasty, total knee arthroplasty, and unicompartmental knee arthroplasty: a systematic review of the literature. JBJS Rev. 2016;4. 10.2106/JBJS.RVW.16.00002 28060788

[9] Charlson M, Szatrowski TP, Peterson J, Gold J (1994). Validation of a combined comorbidity index. J Clin Epidemiol.

[10] Saklad M (1941). Grading of patients for surgical procedures. Anesthesiol J Am Soc Anesthesiol.

[11] Debbi EM, Rajaee SS, Spitzer AI, Paiement GD. Smoking and total hip arthroplasty: increased inpatient complications, costs, and length of stay. J Arthroplast. 2019. 10.1016/j.arth.2019.03.059 31027857

[12] Duchman KR, Gao Y, Pugely AJ, Martin CT, Noiseux NO, Callaghan JJ (2015). The effect of smoking on short-term complications following total hip and knee arthroplasty. J Bone Joint Surg Am.

[13] Halawi MJ, Allen DA, Baron S, Savoy L, Williams VJ, Cote MP (2019). Tobacco smoking independently predicts lower patient-reported outcomes: new insights on a forgotten epidemic. J Arthroplast.

[14] Rosinsky PJ, Chen JW, Lall AC, Shapira J, Maldonado DR, Domb BG. Can we help patients forget their joint? Determining a Threshold for Successful Outcome for the Forgotten Joint Score. J Arthroplast. 2019. 10.1016/j.arth.2019.08.014 31506184

[15] Dindo D, Demartines N, Clavien P-A (2004). Classification of surgical complications: a new proposal with evaluation in a cohort of 6336 patients and results of a survey. Ann Surg.

[16] Clavien PA, Strasberg SM (2009). Severity grading of surgical complications. Ann Surg.

[17] Amanatullah DF, Stryker L, Schoenecker P, Taunton MJ, Clohisy JC, Trousdale RT (2015). Similar clinical outcomes for THAs with and without prior periacetabular osteotomy. Clin Orthop.

[18] Goyal N, Chen AF, Padgett SE, Tan TL, Kheir MM, Hopper RH (2017). Otto Aufranc award: a multicenter, randomized study of outpatient versus inpatient total hip arthroplasty. Clin Orthop.

[19] Brembo EA, Kapstad H, Van Dulmen S, Eide H (2017). Role of self-efficacy and social support in short-term recovery after total hip replacement: a prospective cohort study. Health Qual Life Outcomes.

[20] Quintana JM, Escobar A, Aguirre U, Lafuente I, Arenaza JC (2009). Predictors of health-related quality-of-life change after total hip arthroplasty. Clin Orthop.

[21] Goodman SM, Mehta B, Zhang M, Szymonifka J, Nguyen JT, Lee L (2018). Disparities in total hip arthroplasty outcomes: census tract data show interactions between race and community deprivation. J Am Acad Orthop Surg.

[22] D’Apuzzo MR, Villa JM, Alcerro JC, Rossi MD, Lavernia CJ (2016). Total joint arthroplasty: a granular analysis of outcomes in the economically disadvantaged patient. J Arthroplast.

[23] Clement ND, Muzammil A, Macdonald D, Howie CR, Biant LC (2011). Socioeconomic status affects the early outcome of total hip replacement. J Bone Joint Surg (Br).

[24] Clement ND, Macdonald D, Burnett R, Simpson AHRW, Howie CR (2017). A patient’s perception of their hospital stay influences the functional outcome and satisfaction of total knee arthroplasty. Arch Orthop Trauma Surg.

[25] Hospital Adjusted Expenses per Inpatient Day [Internet]. Henry J Kais. Fam. Found. 2019 [cited 2019 Jun 27]. Available from: https://www.kff.org/health-costs/state-indicator/expenses-per-inpatient-day/.

[26] Berger RA, Sanders SA, Thill ES, Sporer SM, Della VC (2009). Newer anesthesia and rehabilitation protocols enable outpatient hip replacement in selected patients. Clin Orthop.

[27] Klapwijk LCM, Mathijssen NMC, van Egmond JC, Verbeek BM, Vehmeijer SBW (2018). The first 6 weeks of recovery after primary total hip arthroplasty with fast track. Acta Orthop.

[28] Hoffmann JD, Kusnezov NA, Dunn JC, Zarkadis NJ, Goodman GP, Berger RA (2018). The shift to same-day outpatient joint arthroplasty: a systematic review. J Arthroplast.

[29] Kurtz SM, Lau EC, Ong KL, Adler EM, Kolisek FR, Manley MT (2017). Which clinical and patient factors influence the national economic burden of hospital readmissions after total joint arthroplasty?. Clin Orthop.

[30] Courtney PM, Boniello AJ, Berger RA (2017). Complications following outpatient total joint arthroplasty: an analysis of a national database. J Arthroplast.

[31] Shah RP, Karas V, Berger RA (2019). Rapid discharge and outpatient total joint arthroplasty introduce a burden of care to the surgeon. J Arthroplast.

[32] Arshi A, Leong NL, Wang C, Buser Z, Wang JC, SooHoo NF (2019). Outpatient total hip arthroplasty in the United States: a population-based comparative analysis of complication rates. J Am Acad Orthop Surg.

[33] Pincus D, Jenkinson R, Paterson M, Leroux T, Ravi B (2020). Association between surgical approach and major surgical complications in patients undergoing total hip arthroplasty. JAMA..

[34] Aggarwal VK, Elbuluk A, Dundon J, Herrero C, Hernandez C, Vigdorchik JM (2019). Surgical approach significantly affects the complication rates associated with total hip arthroplasty. Bone Jt J.

[35] Jahng KH, Bas MA, Rodriguez JA, Cooper HJ (2016). Risk factors for wound complications after direct anterior approach hip arthroplasty. J Arthroplast.

[36] Purcell RL, Parks NL, Cody JP, Hamilton WG (2018). Comparison of wound complications and deep infections with direct anterior and posterior approaches in obese hip arthroplasty patients. J Arthroplast.

[37] Paxton EW, Cafri G, Nemes S, Lorimer M, Kärrholm J, Malchau H (2019). An international comparison of THA patients, implants, techniques, and survivorship in Sweden, Australia, and the United States. Acta Orthop.

